# Calibration of pedestrian ingress model based on CCTV surveillance data using machine learning methods

**DOI:** 10.1371/journal.pone.0293679

**Published:** 2024-01-18

**Authors:** Martina Pálková, Ondřej Uhlík, Tomáš Apeltauer

**Affiliations:** Faculty of Civil Engineering, Brno University of Technology, Brno, Czech Republic; University of Lagos Faculty of Engineering, NIGERIA

## Abstract

Machine learning methods and agent-based models enable the optimization of the operation of high-capacity facilities. In this paper, we propose a method for automatically extracting and cleaning pedestrian traffic detector data for subsequent calibration of the ingress pedestrian model. The data was obtained from the waiting room traffic of a vaccination center. Walking speed distribution, the number of stops, the distribution of waiting times, and the locations of waiting points were extracted. Of the 9 machine learning algorithms, the random forest model achieved the highest accuracy in classifying valid data and noise. The proposed microscopic calibration allows for more accurate capacity assessment testing, procedural changes testing, and geometric modifications testing in parts of the facility adjacent to the calibrated parts. The results show that the proposed method achieves state-of-the-art performance on a violent-flows dataset. The proposed method has the potential to significantly improve the accuracy and efficiency of input model predictions and optimize the operation of high-capacity facilities.

## Introduction

The development of pedestrian modeling has been ongoing since the 1970s [1] and was originally motivated by the need to analyze the evacuation process of buildings more accurately. Over the last few decades, dozens of evacuation models have been developed and used in performance-based fire safety design [2]. In the field of microscopic models, agent-based models are the most prevalent [3].

The demographic growth of the population has contributed to the need to model the operation of buildings not only in terms of safety but also in terms of comfort of use. Another motivation is undoubtedly the need to optimize the operation of buildings with high attendance, such as hospitals, airports or vaccination centers during pandemics.

One of the biggest challenges of pedestrian modeling is obtaining valid empirical calibration data that describes pedestrian characteristics (number of occupants, their location, walking speed distribution, gender and age distribution, etc.) and behavioral patterns (waiting locations, waiting times, exit choice, etc.). The conventional approach to obtaining this empirical data is to conduct an experiment and measure these quantities or use data from experiments already carried out, such as [4]. For egress modeling tasks, it is necessary to conduct these experiments to obtain key quantities such as pre-evacuation times, densities, evacuation times, etc. Ingress modeling tasks require calibration data from normal occupants’ movement in the object which allows researchers to use data from CCTV monitoring systems. Ingress models can be used for non-evacuation tasks such as capacity assessment, operation analysis and optimization, etc. Some evacuation models are being adapted for potential use in non-evacuation scenarios, for example, the latest versions of Pathfinder [5]. Modern techniques of computer vision, specifically pedestrian tracking algorithms (PTA), allow us to automatically extract some ingress model inputs from mined trajectories without the need for conducting an experiment. This process is called trajectory mining. The main advantages of this approach, from a pedestrian modeling point of view, are:

- the use of unique anonymous data for a specific object and therefore considering local conditions,
- spatiotemporal microscopic analysis of pedestrian trajectories and the possibility to analyze a larger amount of model inputs or validation quantities (interpersonal or pedestrian-object distances, density, flow, usage of space, etc.),
- aggregation of huge volumes of data.

One of the challenges that must be dealt with is the pre-processing of huge volumes of data extracted from video recordings. Raw data generated by PTA are largely noisy due to occlusion, depending on the used PTA, the presence of static objects, height position and tilt of the camera.

The purpose of this paper is to demonstrate the unique concept of using CCTV data for more accurate model calibration. The method for automatic data cleaning of trajectories tracked from video recordings of a COVID-19 vaccination center operation is proposed. A dataset including 408 trajectories was analyzed. The first step was an automatic cluster analysis based on start and end points of trajectories with the aim of distributing the trajectories according to the direction of movement. In a follow-up step, a procedure was proposed to identify noise in the trajectories caused by occlusions and to approximate the missing parts of the trajectories by a straight line. In this task, 9 machine learning classification algorithms were tested. Cleaned data are then processed into descriptive statistics, which can be used as a direct ingress model calibration input. These statistics characterize the walking speed of occupants, distribution of occupants’ waiting points and waiting times. An ingress model of a vaccination center waiting room was designed in Pathfinder software for the purpose of validating the proposed method.

### State of the art

With the massive emergence of artificial intelligence in engineering fields in recent years, a number of publications have emerged that implement machine learning and computer vision methods in the field of pedestrian modeling. Bahamid et al. summarized most of them in his review with conclusions that convolutional neural networks and recurrent neural networks are effective in abnormality detection and prediction. In terms of this paper, the most relevant publications will be mentioned [6]. Johansson et al. deal with the calibration of social force model parameters based on video tracked trajectories [7]. Numerous publications deal with occupant path planning. Yao et al. in [8] propose a reinforcement learning-based data-driven crowd evacuation (RL-DCE) framework. The framework consists of a data-driven crowd evacuation (DCE) model, a cohesiveness-based K-means (C-K-means) algorithm, and a hierarchical path planning mechanism. Wang et al. in [9] introduce the improved multi-agent reinforcement learning method (IMARL algorithm) for path planning-based crowd simulation. The IMARL algorithm improves the convergence speed and evacuation efficiency and provides specific guidance schemes for crowd evacuation improvement and assists in decision support for the prevention and management of large-scale group trampling incidents. A deep deterministic policy gradient algorithm for path planning in crowd evacuation scenarios is proposed by Li et al. in [10]. The algorithm utilizes reinforcement learning and deep neural networks to find optimal evacuation paths that maximize the safety of evacuees. The authors test their algorithm on various scenarios and compare its performance to other path planning algorithms. The results show that the proposed algorithm outperforms other approaches in terms of safety, efficiency, and scalability. In [11], Kim et al. propose a socially adaptive path planning system for navigating robots in human crowds using inverse reinforcement learning. The authors demonstrate the effectiveness of their approach through experiments with a wheelchair robot in simulated environments.

Real-time anomaly detection is also studied. In [12], Alsalat et al. propose a system that utilizes machine learning and internet of things (IoT) devices to detect and prevent mass panic. Their system collects data from IoT devices, such as cameras and heart rate sensors to monitor the behavior of individuals in a crowd. Machine learning algorithms are then used to analyze this data and detect any signs of panic, such as increased heart rate and rapid movements. Yuan et al. [13] propose a method for detecting anomalies based on the analysis of the structure of the crowd. They used computer vision techniques to extract features from the video footage of the crowd, such as motion trajectories, spatial layout, and the relationships between individuals in the crowd. The extracted features were then used to construct a graph representing the structure of the crowd. Anomalies were detected by analyzing the graph and identifying nodes or edges that deviate significantly from the normal patterns of the crowd’s behavior. The proposed method was tested on real-world video footage, and the results showed that it outperformed other state-of-the-art anomaly detection methods. Marsden et al. [14] introduces a new method for detecting crowd behavior anomalies using a combination of four crowd features: crowd collectiveness, crowd conflict, mean walking speed, and crowd density. Two different anomaly detection approaches are tested, one using a Gaussian Mixture Model (GMM) for outlier detection with only normal training data available, and the other using a Support Vector Machine (SVM) for binary classification with both normal and abnormal training data available. The method achieves state-of-the-art classification performance on a violent-flows dataset. Guo et al. in [15] present a method for detecting and localizing anomalies in crowded scenes using short-term trajectories. Their approach involved analyzing the motion patterns of individuals in a crowd to identify abnormal behaviors that may indicate an emergency situation. Chaker et al. in [16] propose a social network-based approach for detecting and localizing anomalies in crowds. Their method utilized the connections between individuals in a crowd to build a social network. The authors evaluated their method on two datasets and achieved high accuracy in both anomaly detection and localization.

Goldhammer et al. in [17] proposed the use of artificial neural networks (ANNs) for predicting pedestrian movements in public traffic. The authors developed an ANN-based model that can accurately forecast the future short-time trajectories of pedestrians. Schulz et al. in [18] proposed a method for pedestrian intention recognition and path prediction within driver assistance systems, which utilizes a controlled interactive multiple model filter. This approach integrates multiple sensor modalities and fuses their outputs to estimate the pedestrian’s position and intention. The proposed method achieves high accuracy in predicting pedestrian motion and intention.

#### Trajectory mining

It is still a challenge to effectively calibrate and validate agent-based models [19]. One option is to use trajectories and data of pedestrian movement in general. These can now be easily obtained in many ways and in nearly real-time. This is facilitated, for example, by GPS, Wi-Fi technologies or radio frequency identification (RFID) [20,21] as well as the widespread use of security cameras in public areas. It is possible to obtain trajectories from their records, for example, using machine learning methods. In this way, huge databases of pedestrian movements can be obtained very quickly, but they must be effectively analyzed in bulk. Trajectory mining deals with this issue. It includes, for example, clustering, classification, prediction, pattern mining or outlier detection. In the field of pedestrian dynamics, Karim used GPS data from pedestrians’ mobile devices for modeling risks to which pedestrians are exposed [22]. Wei deals with the reconstruction of trajectories in urban areas with small sampling frequency [23]. Drira tracked occupant trajectories using floor-vibration measurements [24,25].

#### Trajectory clustering

Clustering is an effective way of sorting data into groups based on their similarity. In the case of trajectories, it is possible to perform clustering for entire trajectories or their segments, where the input can be static characteristics (start/end, total time, etc.) or spatiotemporal sequences of data of different lengths, which contain additional metadata, such as the ID of a moving object or timestamp. In this procedure, it is crucial to choose a suitable method for calculating similarity (distance) [26]. Clustering algorithms can be divided into 5 categories [27] according to what is the main criterion for calculating similarity. These categories of algorithms are spatial-based, time-dependent, partition and group-based (for trajectory division), uncertainty between data points and semantic trajectory clustering.

#### Noise detection

Trajectories often contain erroneous points due to the inaccuracy of measuring instruments (GPS) or evaluation algorithms (convolutional neural network for detecting a moving object). Anomalies are samples that differ significantly from the rest but are real and may contain important information (medical diagnosis of an uncommon serious illness). Noise, on the other hand, are samples that are not real and were caused by an error in measuring or pre-processing of data. Noise can significantly negatively affect predictions and skew the results. For these reasons, noise detection is an important topic in many fields.

There are different approaches to determining noise in trajectories. Some divide trajectories into segments with similar properties, and these segments are further processed [28]. Others, on the other hand, treat individual data points as separate elements and classify them. The classification of segments or points can then take place, for example by methods of supervised learning—supervised learning (classification) [29], unsupervised learning (clustering) [30] or semi-supervised [31].

## Methods

The following subsections describe the data and methods used to filter and clean the trajectories.

### Data

The data was acquired by an automatic pedestrian traffic detector that uses a convolutional neural network and a tracking algorithm to store anonymous trajectories of pedestrian movements based on real-time monitoring. Data from one camera at the high-capacity vaccination center waiting room in Brno, Czech Republic, was used for the analysis. Data includes trajectories taken between 7:00 AM and 7:30 AM, just after the center opened. Detection was conducted through real-time monitoring using a Hikvision network camera with a 1080p resolution and 25 fps frame rate. The camera’s attributes employed for the measurements align with those commonly utilized in practical applications. The trajectories were smoothed in a post-processing phase. The dataset includes instantaneous and average walking speed, slope (the angle between the horizontal line and the connecting line with the previous point), lateral and tangential acceleration.

### Framework

**Fig 1:**
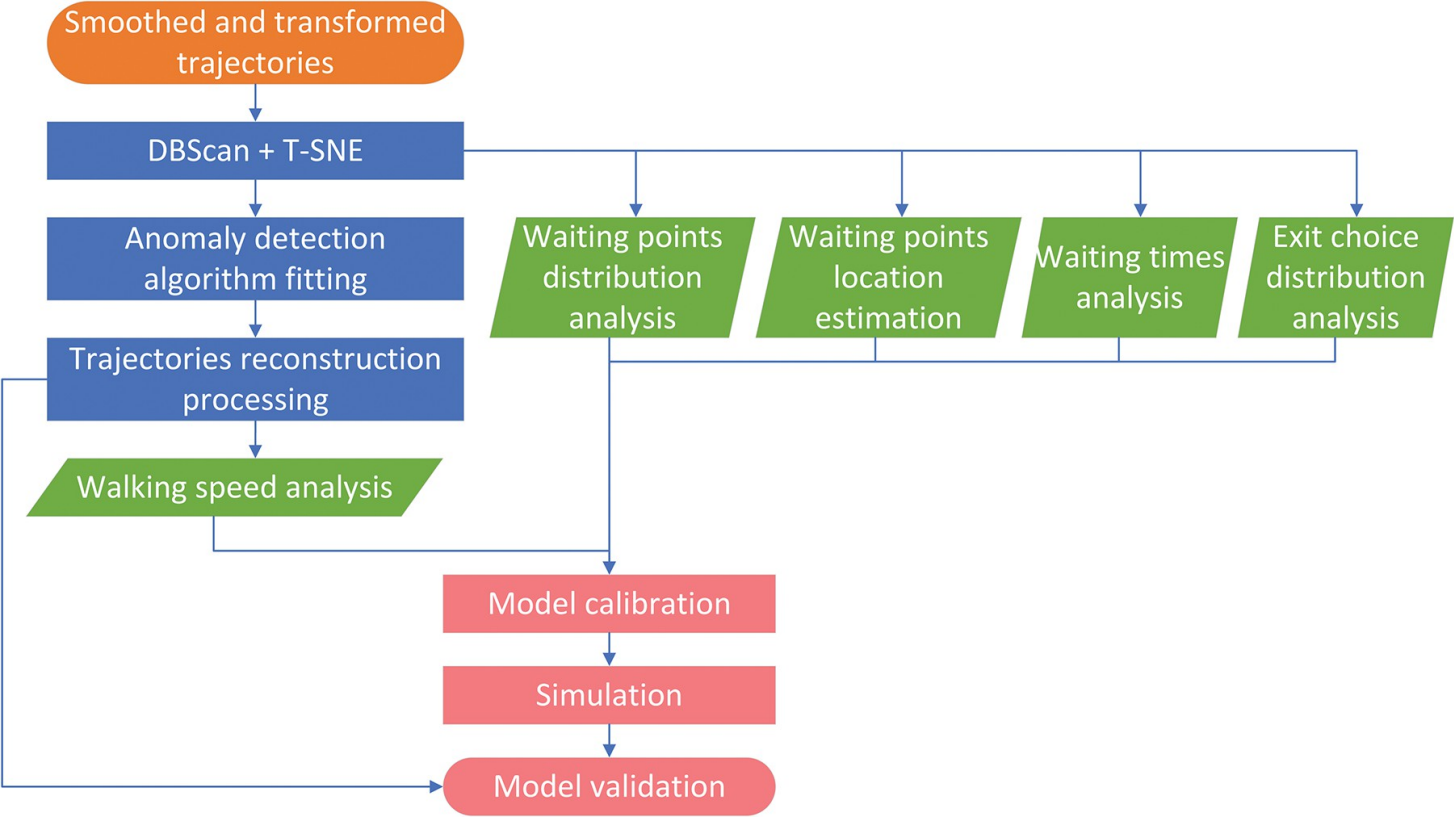
Trajectories processing procedure with their use in agent-based pedestrian model.

The process of extracting pedestrian model parameters is shown in Fig 1. Based on the extracted dataset, clustering analysis is performed to distinguish the trajectories of clients and staff. At the same time, the number of waiting points, their distribution within the population, the distribution of waiting times and the distribution of exit choices is calculated from the ingested dataset. The next step is noise analysis and trajectory processing. Based on the cleaned trajectories, the distribution of walking speed is further estimated.

### Machine learning methods

The following subsections describe the machine learning methods and techniques used.

#### DBSCAN

Density-based spatial clustering of applications with noise (DBSCAN) was introduced by Ester, Hans-Peter Kriegel, Jörg Sander and Xiaowei Xu in 1996 [32]. The samples are understood as points of Euclidean space and clusters are areas of this space with a high density of points. It is therefore not necessary to determine the number of clusters in advance. Points in low-density areas are marked as noise. The advantage of this algorithm is its ability to solve non-convex problems and noise detection [33].

It is necessary to determine two constants for this method—Eps and MinPts. Eps determines the maximum distance between two points (i.e., the larger the value, the smaller the number of clusters). This parameter can be determined using the k-distance graph [34], which shows the distance relationship and k = minPts-1 to the nearest neighbors. The optimal value of Eps is located in the “elbow” of this curve. No procedure is specified for the MinPts option, and this parameter must be estimated with respect to the number of dimensions and properties of the dataset.

#### T-SNE

The t-Distributed Stochastic Neighbor Embedding (t-SNE) method was used to visually check the results of clustering analysis [35]. This method is commonly used to reduce dimensionality (commonly to 2D). It uses a nonlinear transformation algorithm. It is usually necessary to specify two parameters: the number of iteration steps and perplexity. Both constants must be determined empirically. Perplexity can be seen as k in the k-nearest neighbor method.

#### Random forest

Random forest is a supervised ensemble machine learning method used for classification and regression problems. RF is made up of a group of decision trees trained on training data subsets. These subsets are selected by bootstrapping (random selection with replacement). A new training subset is created for each tree. This process is called bootstrap aggregating, or bagging. The results of the individual trees are combined and the most frequently predicted class is determined as the model output. This procedure significantly reduces the generalization error and reduces overfitting. The properties of the model increase with the number of decision trees. Unlike individual trees, the RF method is harder to interpret and thus, as with artificial neural networks, there is a "black box problem"[36].

#### Handling an imbalanced dataset

Data that contains significantly higher proportions for some classes than others is often referred to as unbalanced [37]. After training, an unreliable model is created, which classifies the majority class with high accuracy and the minority class with very little accuracy. In most cases, however, we need high reliability in determining the minority class (tax fraud, disease detection, spam filtering). In our research, we compared two methods used to balance a training dataset. On the other hand, the test dataset should capture the true form of the data as best as possible. This is also one of the reasons why it is recommended to use more complex metrics than the accuracy score, which can be misleading in such cases.

#### Undersampling

If there is a sufficient number of samples in the minority class, the usual procedure is to select a random subset of the majority class that corresponds to the size of the minority class. The disadvantage of this procedure is the possible loss of important information about the relationship between the classes.

#### Data generation–SMOTE

SMOTE is a method of generating random synthetic data for a minority class [38] to rebalance a training dataset. For random points of the minority class, the k-Nearest Neighbors within the class are determined and a new synthetic point is placed along the lines connecting these points. The main shortcomings of this method include minority overgeneralization [39] and noise oversampling [40].

#### Pseudo labelling

Pseudo Labelling is from the family of Semi-supervised learning methods that uses a small amount of labelled data and a large amount of unlabelled data. The method is usually performed in five steps:

1. Training a regressor.
2. Predicting the output value for unlabelled data that was not used for training.
3. Compiling a new training set including newly tagged data that had an output value from the model less than 0.00001 (for class 0) or higher than 0.99999.
4. Training a new model for classification using a new training set.
5. Classifying new data.

#### Perspective transformation

**Fig 2:**
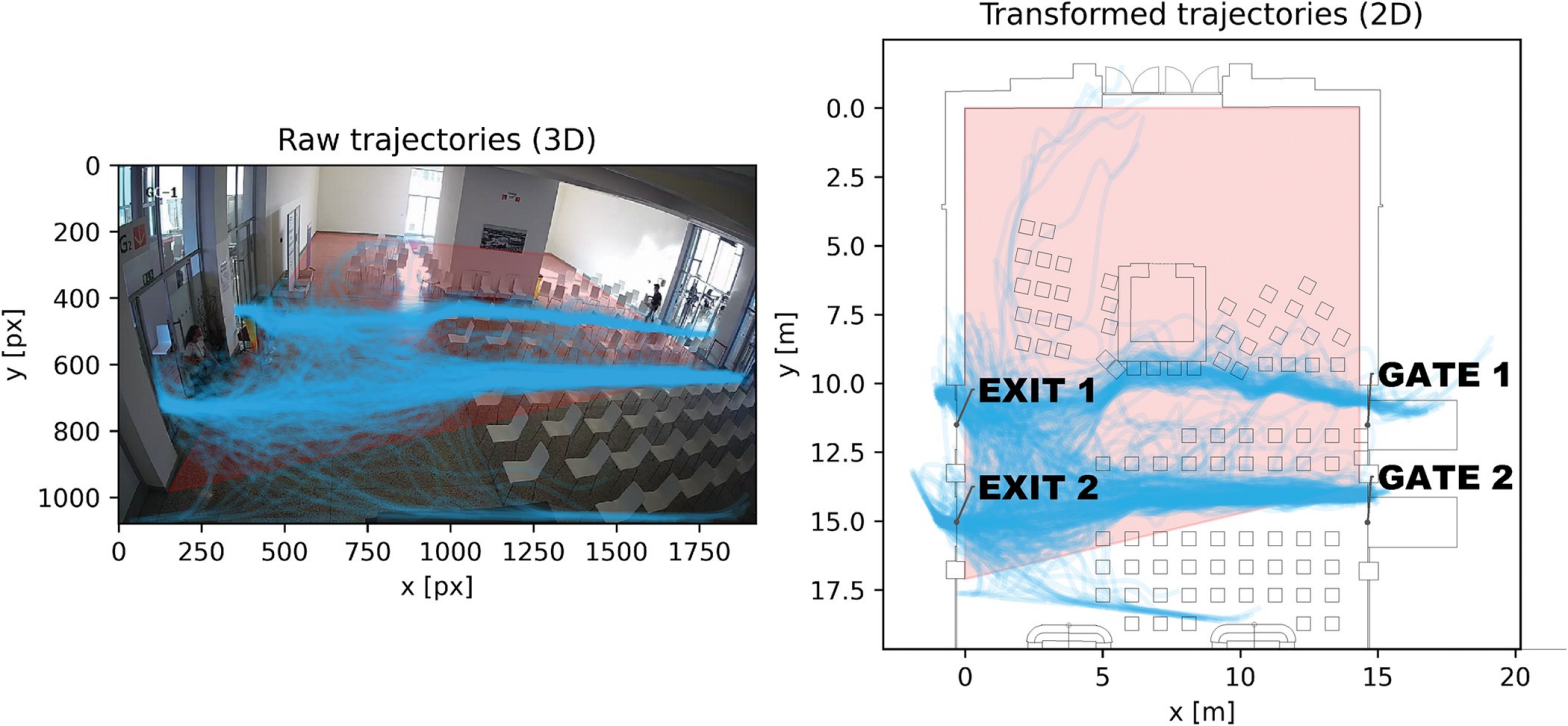
Raw trajectories of vaccination center waiting room with red polygon for transformation matrix estimation (left) and perspective transformation of trajectories (right).

The dataset contained 408 trajectories of several types: occupants passing from entrance to exit, occupants going in the opposite direction, occupants entering, turning and leaving. Raw trajectories were transformed from 3D pixel coordinates to a local 2D coordinate system. Results are presented in Fig 2. A transformation matrix and a polygon bounded by 4 points were used for transformation (red area in Fig 2).

#### Clustering analysis

**Fig 3:**
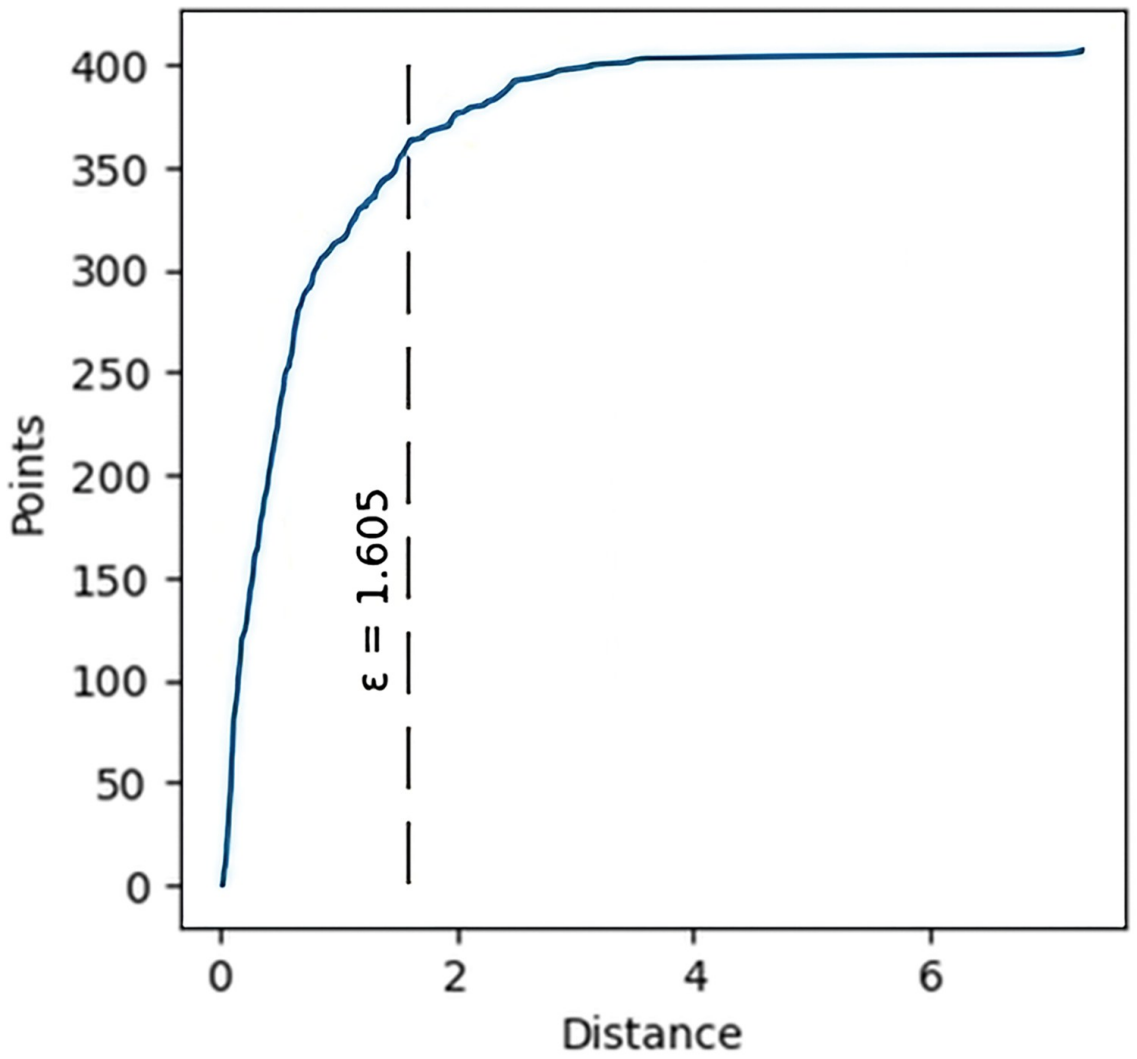
K-distance graph for estimation of optimal value of Eps.

To validate agent simulations, it is necessary to select only those trajectories that belong to persons who are going in the right direction. For these reasons, clusters were analyzed according to the start and end point of each trajectory. The length or similarity of trajectories was not considered because we also need to capture occupants who are waiting and walking around the room. The optimal value of Eps was determined using a k-distance graph. The result can be seen in Fig 3; the value was determined to be 1,605. MinPts was estimated as 10 samples.

**Fig 4:**
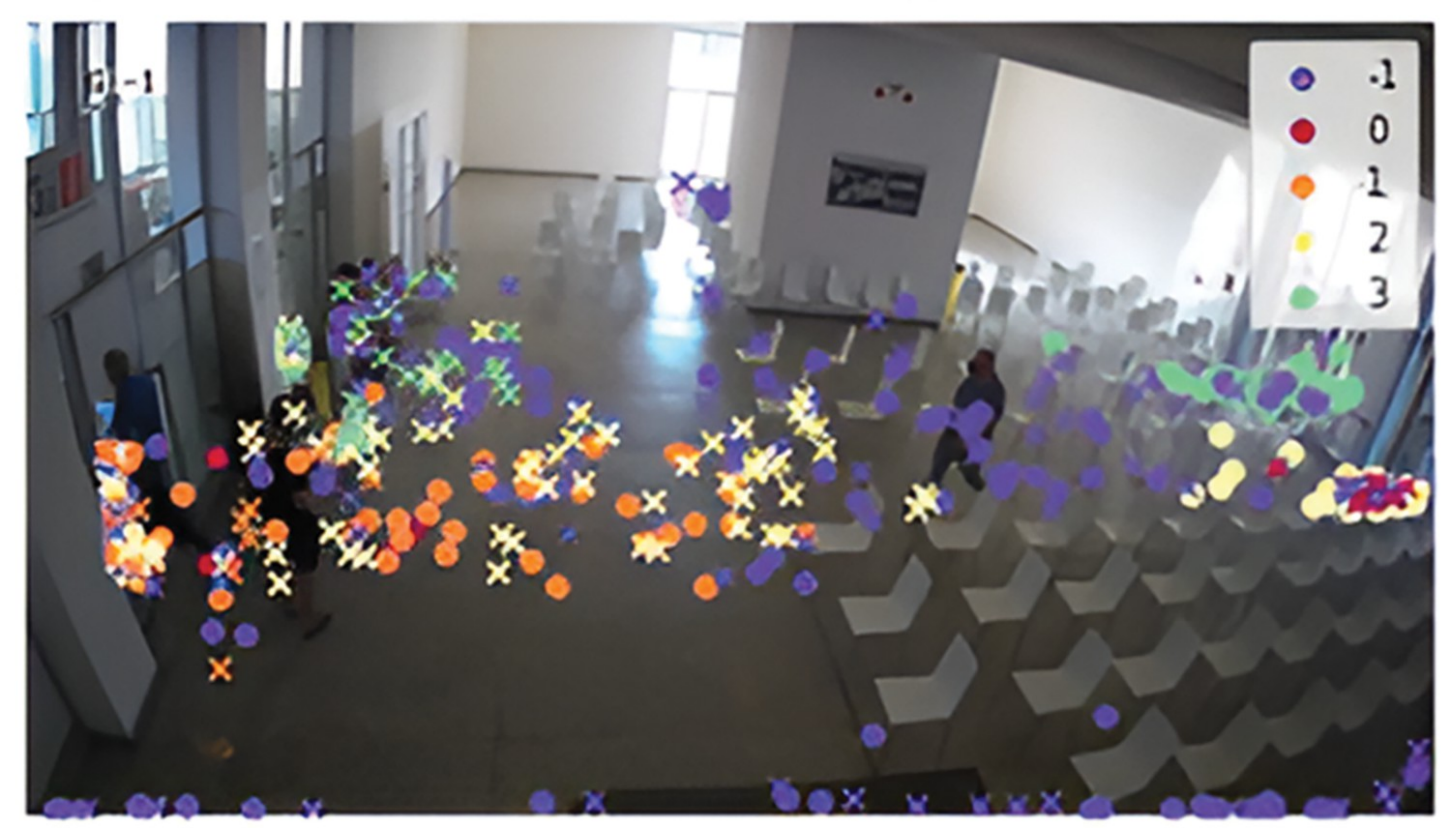
Clustering of start (dot) and end points (cross) of all 408 trajectories.

**Fig 5:**
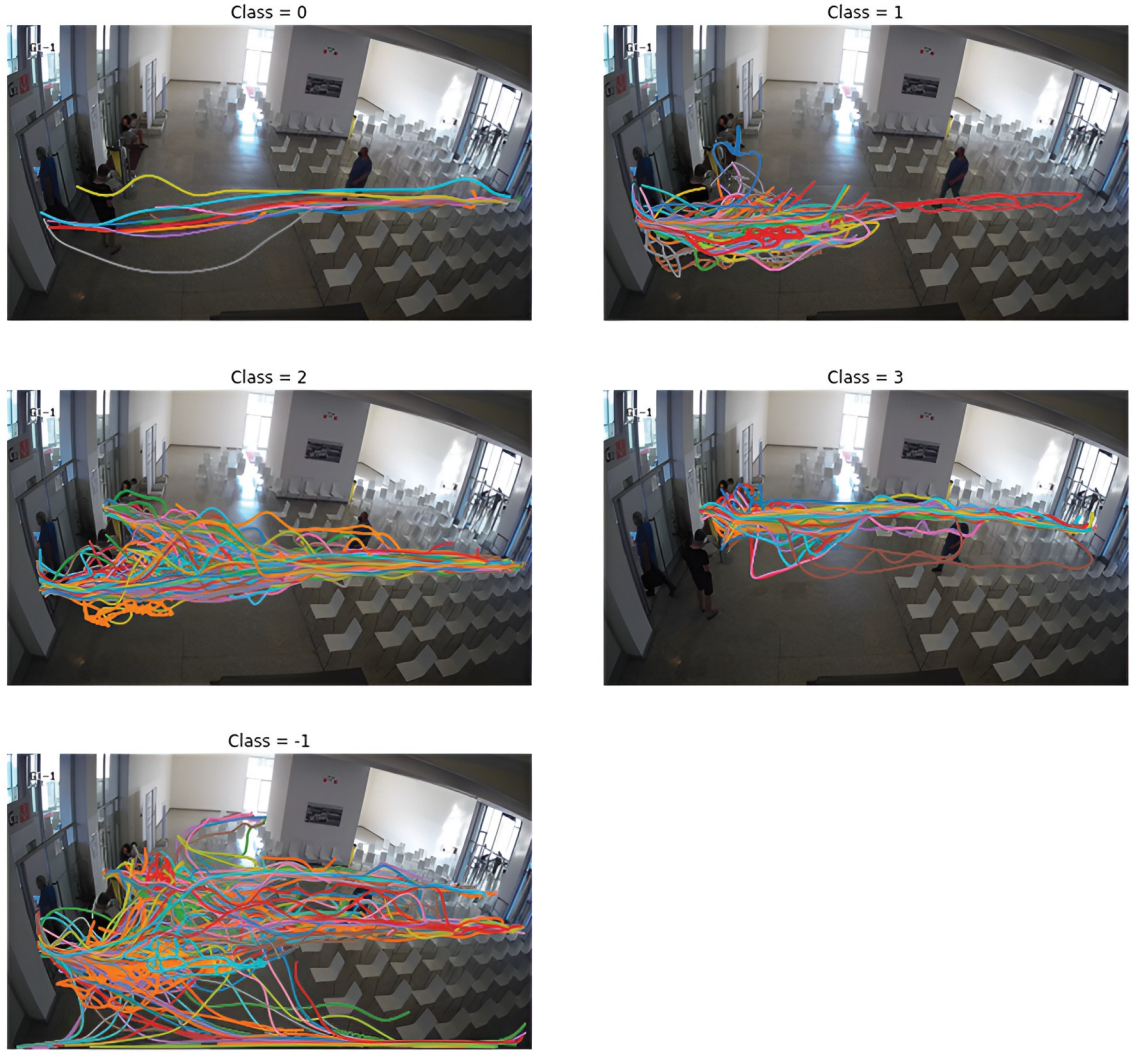
Data visualization for individual classes.

The data were divided into four groups and outliers that did not belong to any group were marked as noise (class -1). The classification of start and end points into clusters is shown in Fig 4. The trajectories belonging to individual classes can be seen in Fig 5. Class 0 indicates occupants walking in the opposite direction (outwards from the vaccination center). Class 1 represents persons staying at the exit, mostly medical staff who check the validity of the center’s client documents. Classes 2 and 3 are occupants who come through one of the two entrances and continue on to the vaccination center. These are the occupants of our interest. There were 144 trajectories in class 2, 82 in class 3, and 128 trajectories were marked as noise.

**Fig 6:**
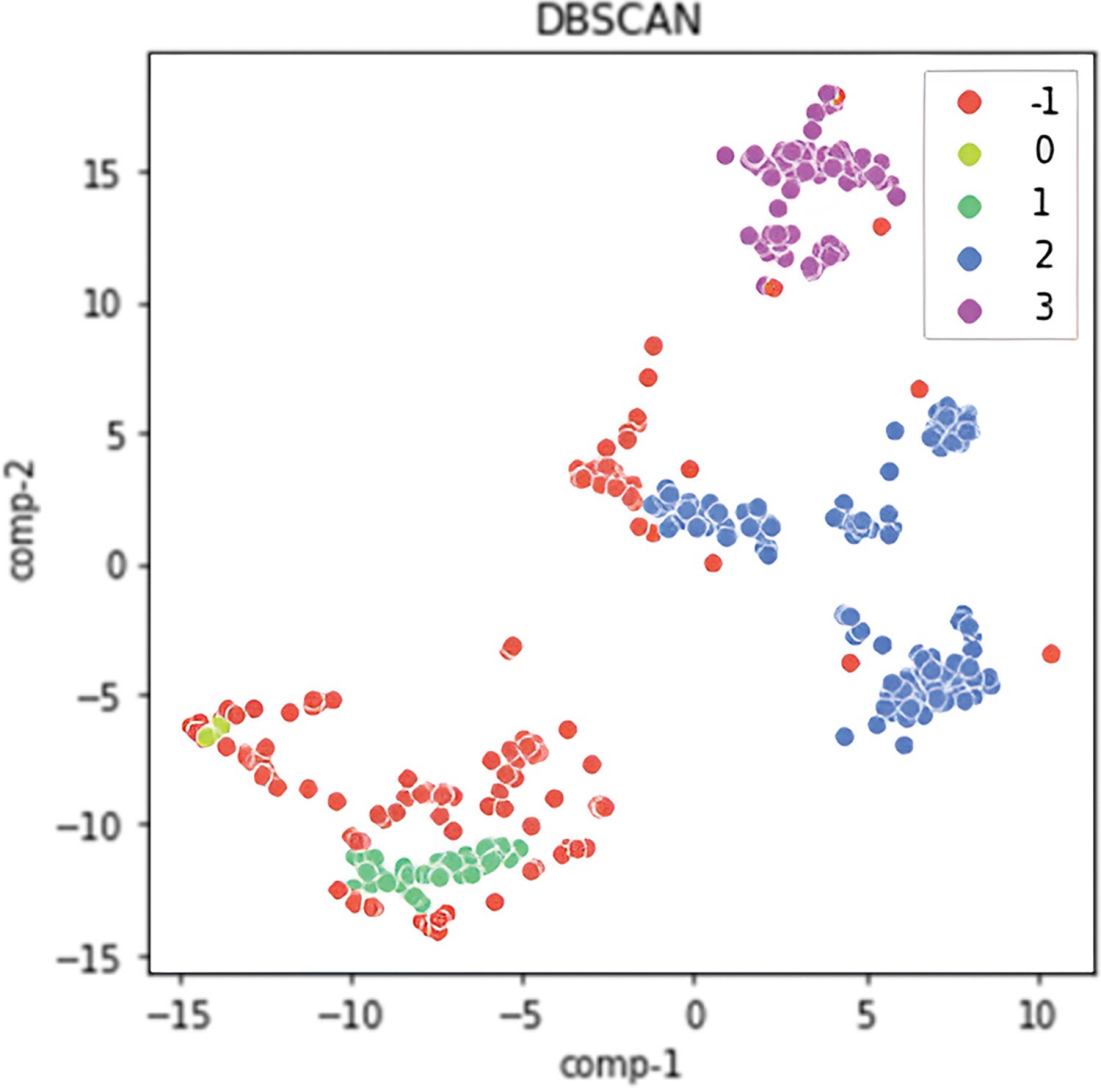
Visualization of cluster analysis results with t-SNE.

For visual inspection, the results of the clustering analysis were displayed using the t-SNE method, which reduced the four-dimensional point space to two-dimensional. The t-SNE graph is shown in Fig 6.

#### Training and testing dataset design

**Fig 7:**
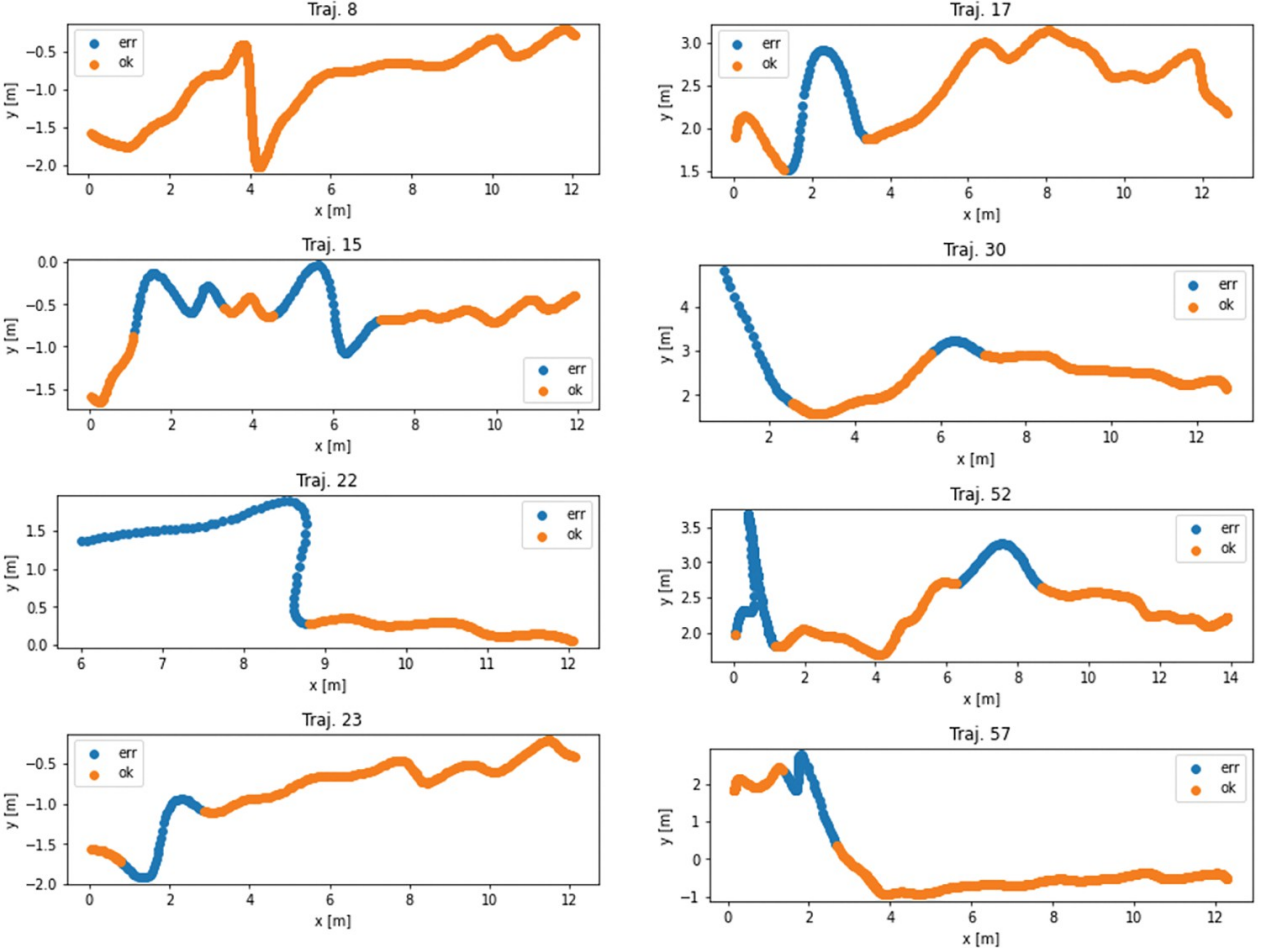
Trajectories from training dataset.

Trajectories from classes 2 and 3 that correspond to occupants walking in the direction of the vaccination process are subsequently analyzed and cleaned using machine learning methods (226 trajectories in total). The training set was compiled in such a way that four trajectories from group 2 and four trajectories from group 3 were randomly selected. Each point of these trajectories was visually marked according to camera records, whether it is wrong or not. A training set was compiled from the points marked in this way (Fig 7). However, because this set can be expected to contain fewer erroneous points (3597 correct points in class 0 (negative) and 712 erroneous in class 1 (positive)), the resulting training dataset was compiled to be balanced.

**Fig 8:**
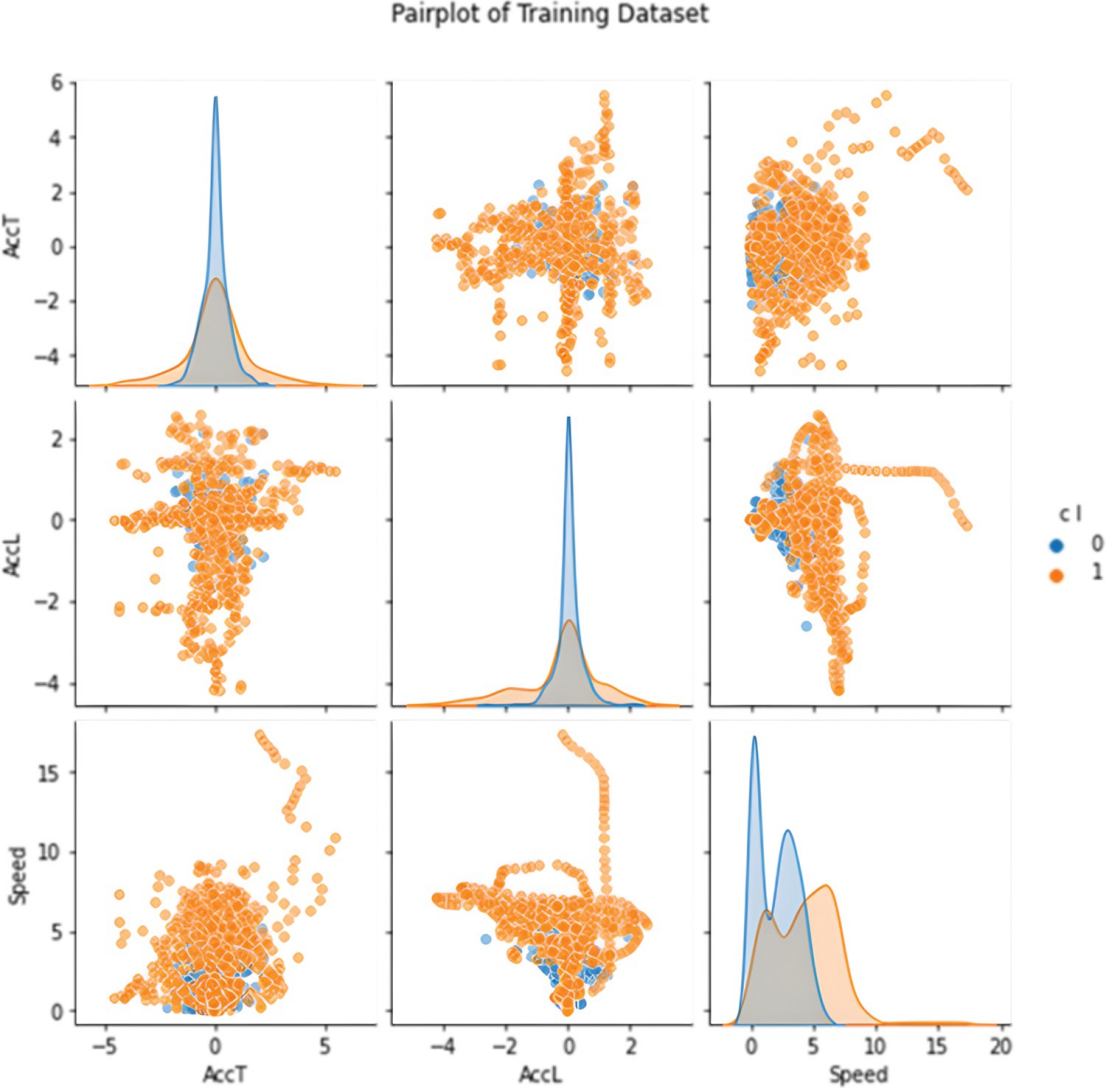
Pairplot of downsampled training dataset.

**Fig 9:**
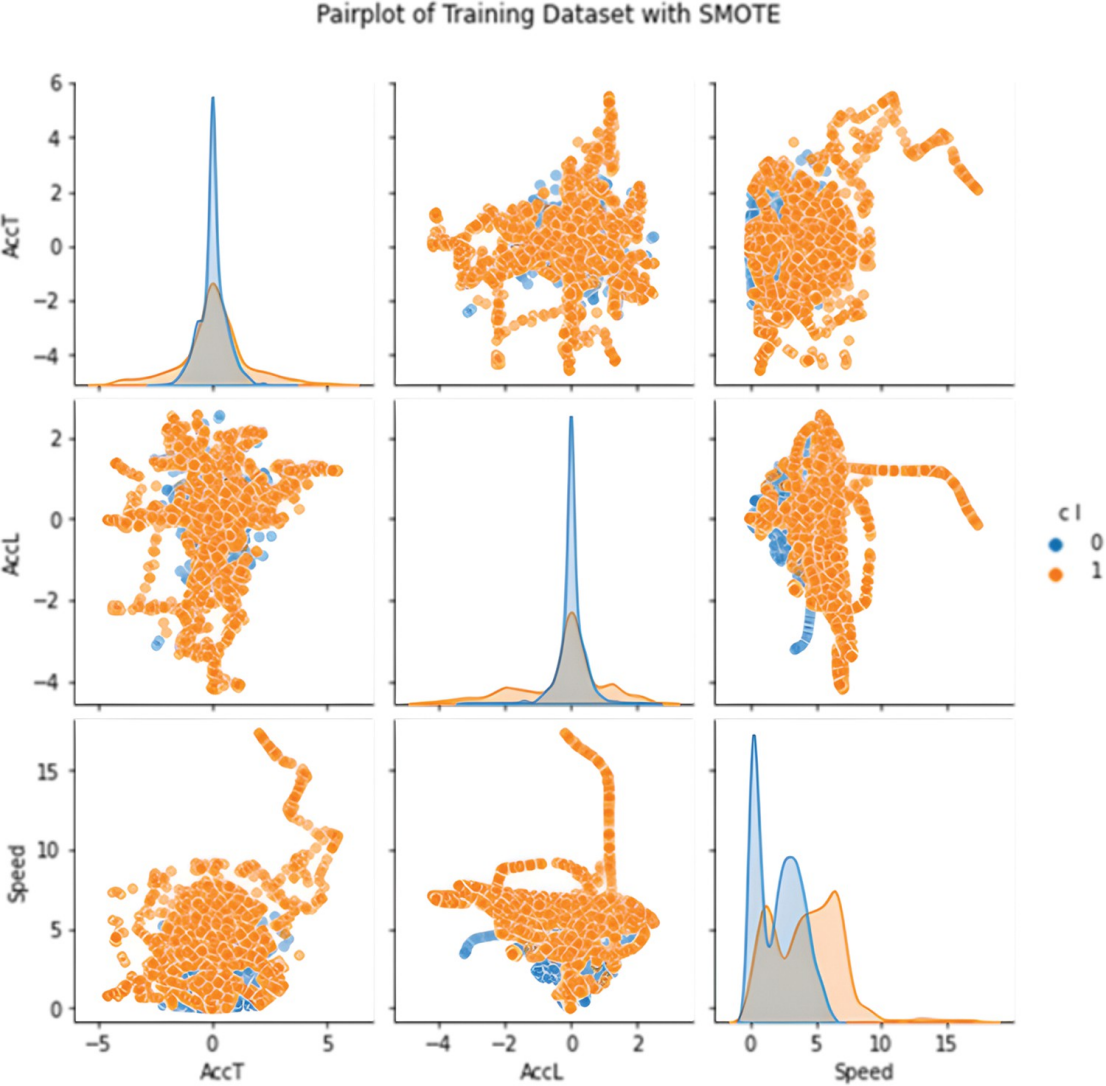
Pairplot of SMOTE training dataset.

**Fig 10:**
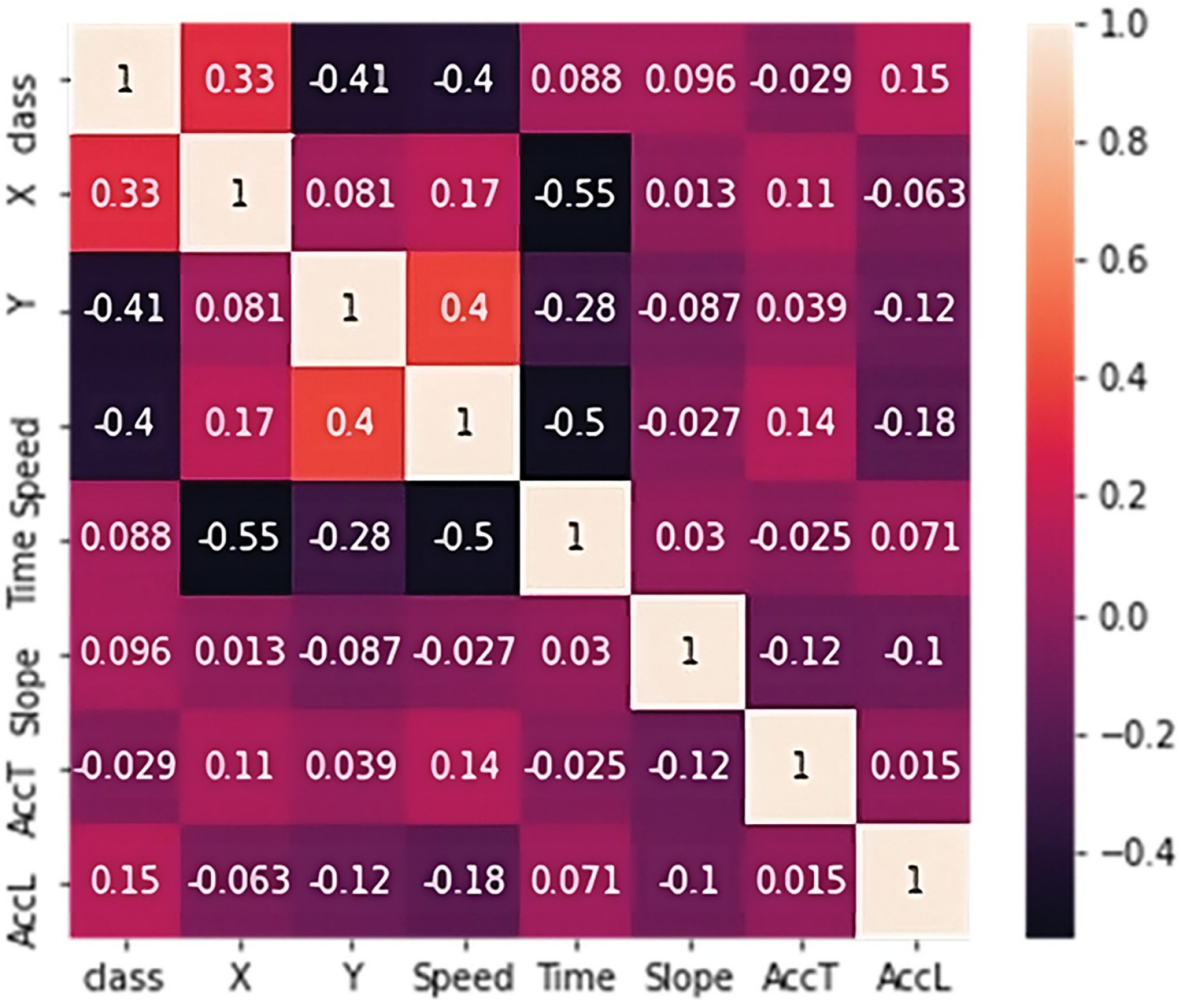
Covariance matrix of training data set.

In the first case, undersampling was performed, so the number of correct points was chosen according to the number of erroneous ones. In this case, the balanced training dataset contained a total of 1424 points, which evenly represented both classes (Fig 8). In the second case, synthetic data for the minority class were generated by the SMOTE method. The training dataset thus contained 7194 points (Fig 9). The sensitivity analysis shows that the class is most affected by coordinates and speed. In an effort to create the most general classifier possible, only speed and both accelerations entered training because error points are more common at exits due to crowding of occupants. However, error points around entrances are just as serious and the model needs to classify them with the same importance as those at exits. The covariance matrix of training set is shown on Fig 10.

Three more trajectories were selected for the test dataset. The dataset contained 1171 points, of which 258 were incorrect (class 1). Unlabeled data then formed another 93172 points.

#### Comparison of classification methods

To select the most suitable ML algorithm, several methods were compared using a ten-fold cross-validation without any tuning of their hyperparameters. Accuracy was chosen as a metric for comparing models; results are shown in Table 1.

**Table 1:** Comparison of classification algorithms.

Algorithm	Accuracy
**Nearest Neighbors**	0.89 (+/- 0.05)
**Linear Support Vector Machines**	0.82 (+/- 0.07)
**RBF Support Vector Machines**	0.89 (+/- 0.06)
**Gaussian Process**	0.89 (+/- 0.06)
**Decision Tree**	0.89 (+/- 0.04)
**Random Forest**	0.90 (+/- 0.07)
**Neural Network**	0.87 (+/- 0.07)
**AdaBoost**	0.87 (+/- 0.08)
**Naive Bayes**	0.88 (+/- 0.07)
**QDA**	0.88 (+/- 0.07)

**Fig 11:**
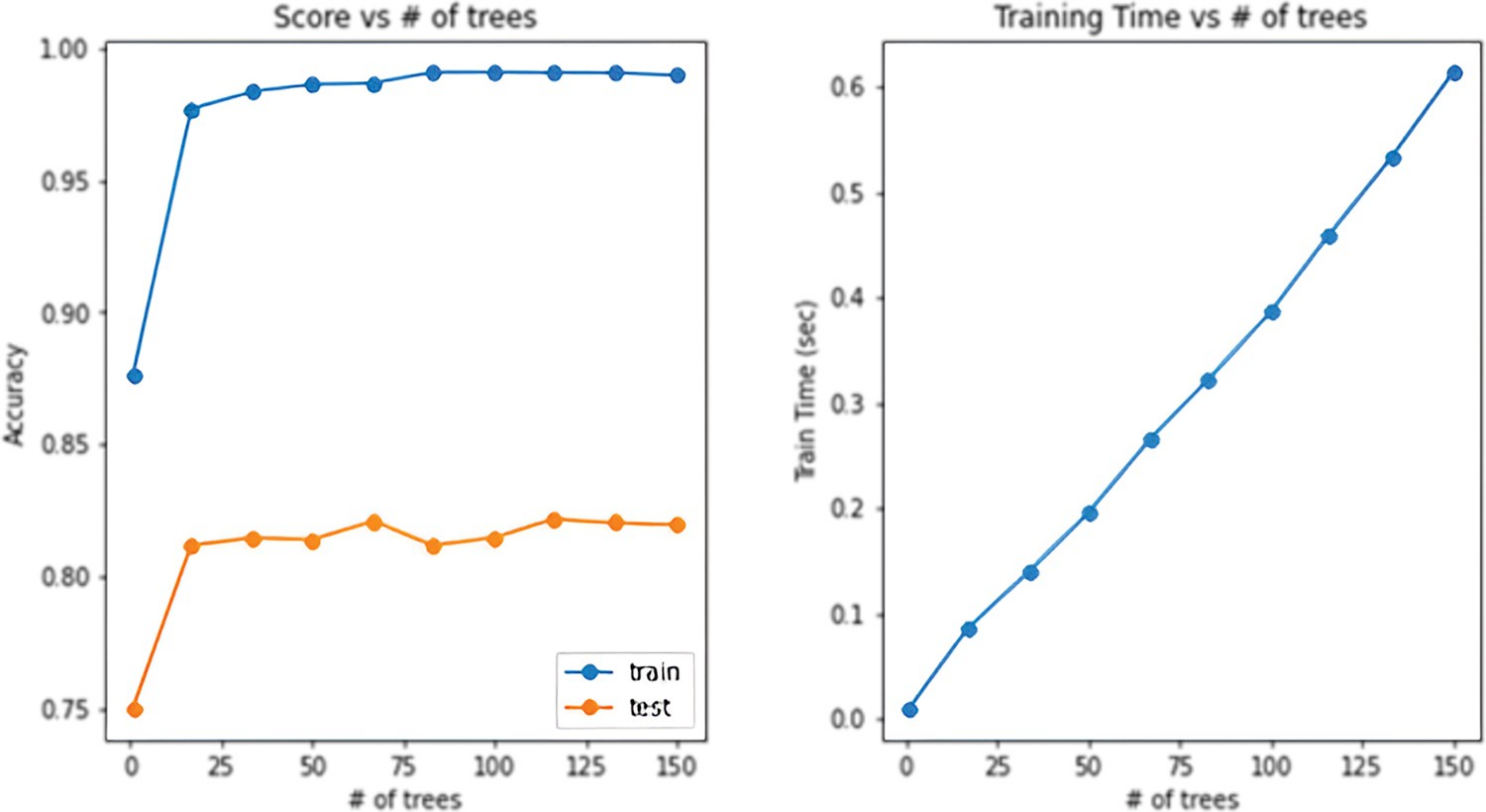
Graph of the dependence of accuracy and training time on the number of trees.

The Random Forest algorithm is then applied based on the Table 1. After performing hyperparameter tuning, the final model consisted of 120 trees. Additional parameters are described in Table 2 and the training process is shown in Fig 11.

**Table 2:** Parameters of random forest classifier.

Feature	Value
**Number of trees**	0.8360
**Max. depth of trees**	0.7626
**Number of features**	0.8471
**Min. samples required to be at leaf node**	1
**Min. samples required to split**	5
**Criterion**	Gini impurity

#### Undersampling train set with pseudo labelling

**Fig 12:**
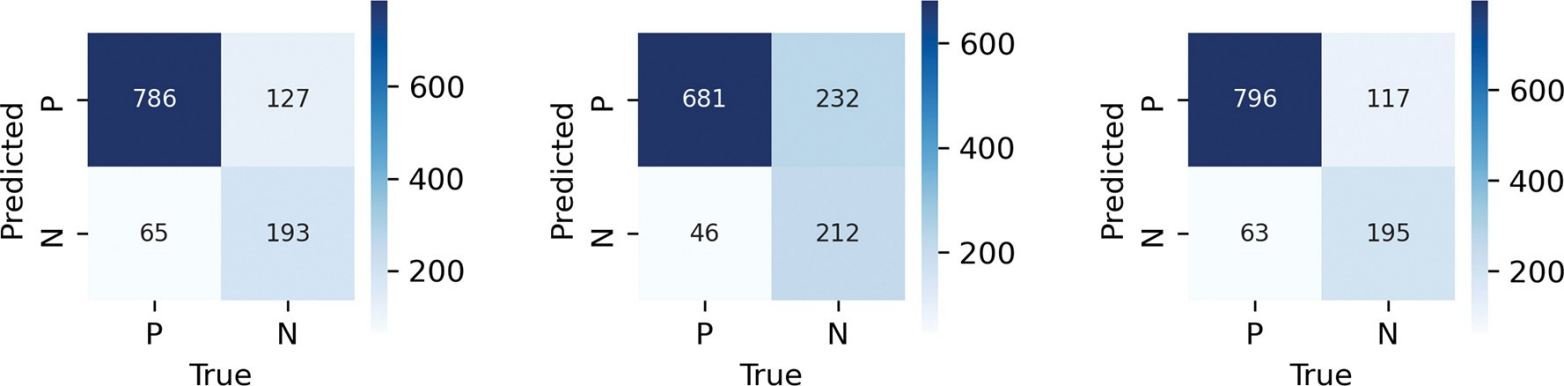
Confusion matrix of undersampled train set (left) with accuracy: 0.836 and recall: 0.7481, undersampled trainset mixed with pseudo labelled data 90% (middle) accuracy: 0.7626 and recall: 0.8217 and undersampled trainset mixed with pseudo labelled data 99% (right) with accuracy: 0.8463 and recall: 0.7558.

In this case, the model was trained on a US dataset and used to classify unlabelled data. If the model determined a class with more than 90% (or 99%) probability, the sample was actually classified and added to the training dataset. The resulting dataset was rebalanced by the undersampling method and 14,830 samples were used for further training. When the probability limit was increased, the new training dataset contained only 3,766 samples. The Fig 12 depicts a confusion matrix of the classifier.

**Fig 13:**
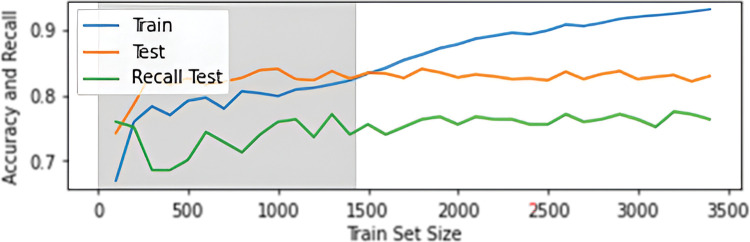
Graph of accuracy score (or recall score) dependence on the size of the training set. The gray area shows when only data from the original training set was used. The accuracy score for the training set was determined by 10-fold cross validation.

It can be seen from Fig 13 that the accuracy of the model on test data decreases when using pseudo-labelled data, but the recall value increases and stabilizes; on the contrary, the accuracy of training data (determined by ten-fold cross-validation) increases. This phenomenon is a form of overfitting and is called confirmation bias [41]. The model is essentially consolidating its truth, reducing its ability to generalize; this is even more apparent when increasing the probability (to 99%) limit for classifying a sample.

#### SMOTE

**Fig 14:**
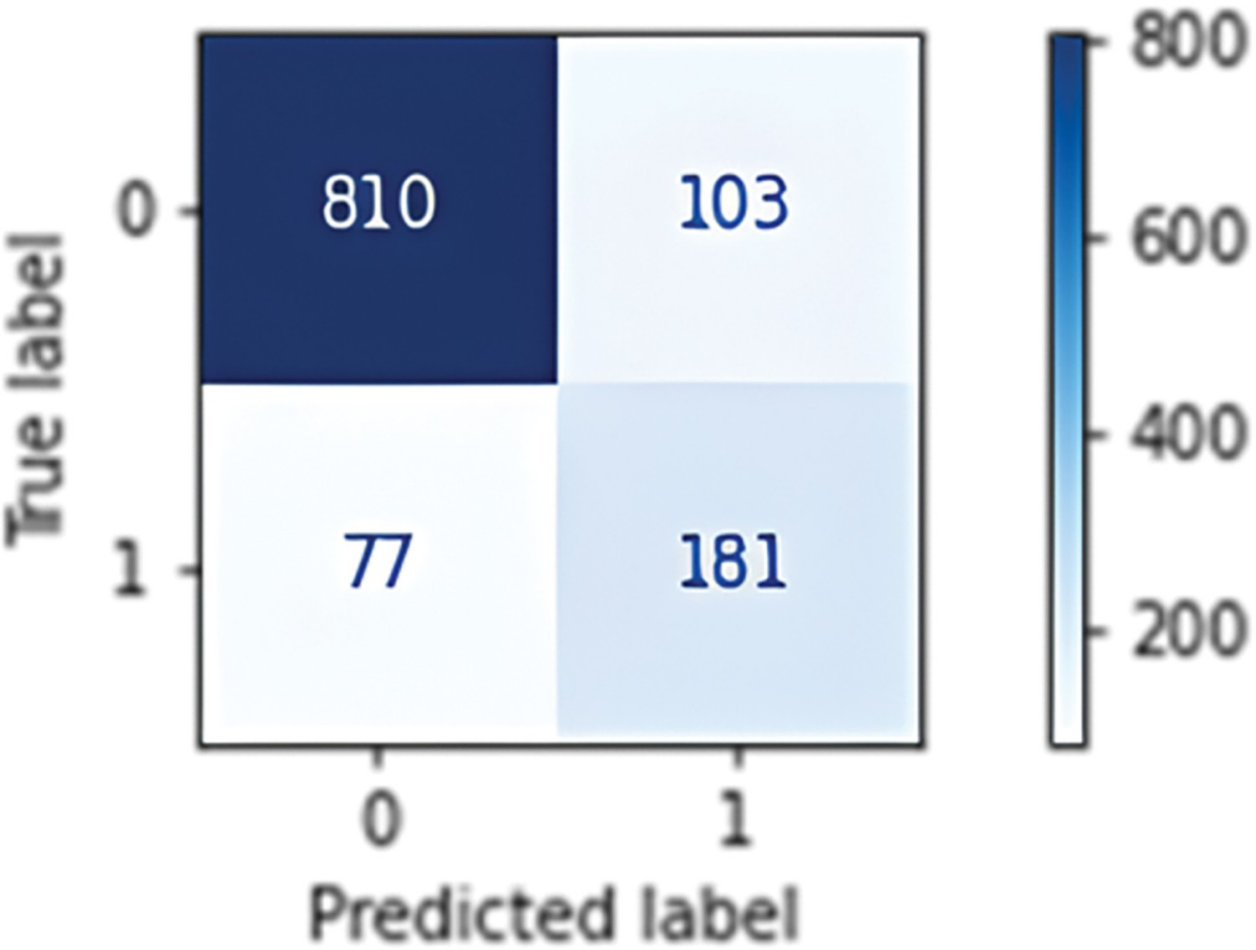
Confusion matrix of the model using SMOTE data set with accuracy: 0.8471 and recall: 0.7016.

**Fig 15:**
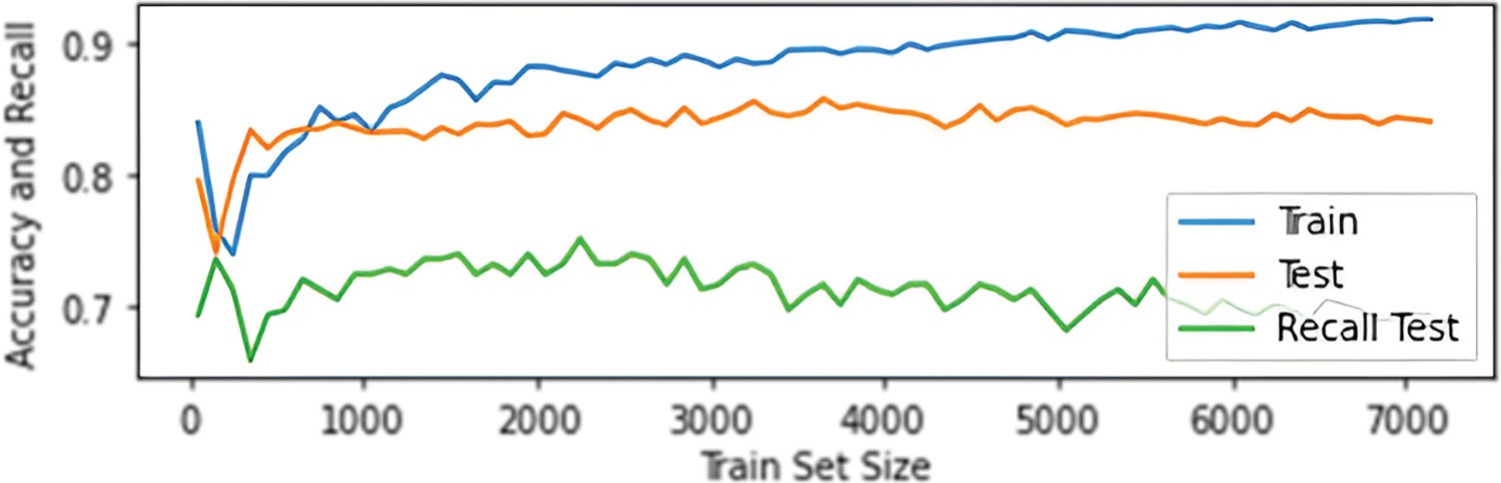
Graph of the dependence of accuracy and recall scores on the size of the training set. The accuracy score for the training set was determined by 10-fold cross-validation.

The use of the SMOTE method increased the accuracy of the model on test data, but at the same time reduced the Recall score; this phenomenon is probably due to oversampling of non-informative samples; this is evident, for example, in changes in speed distribution, see Fig 14. The Fig 15 illustrates the relationship between accuracy and recall based on the size of the training set.

#### Comparison of models

For purposes of our research, it is more important to correctly determine error points; thus, a type one error is not as significant as a type two error; for this reason, Recall (Sensitivity) metric was chosen to compare resulting models, which describes model’s ability to predict positive output; according to this metric, most suitable model is combination of US and PS datasets. The results of the comparison can be seen in Table 3.

**Table 3:** Results of model comparisons.

Train Set	Test Accuracy	F1	Recall
**US**	0.8360	0.675	0.7481
**US and Pseudo Labelling (90%)**	0.7626	0.604	0.8217
**SMOTE**	0.8471	0.669	0.7016

## Results and discussion

The following subsections describe the results obtained using the above methods.

### Trajectories cleaning

**Fig 16:**
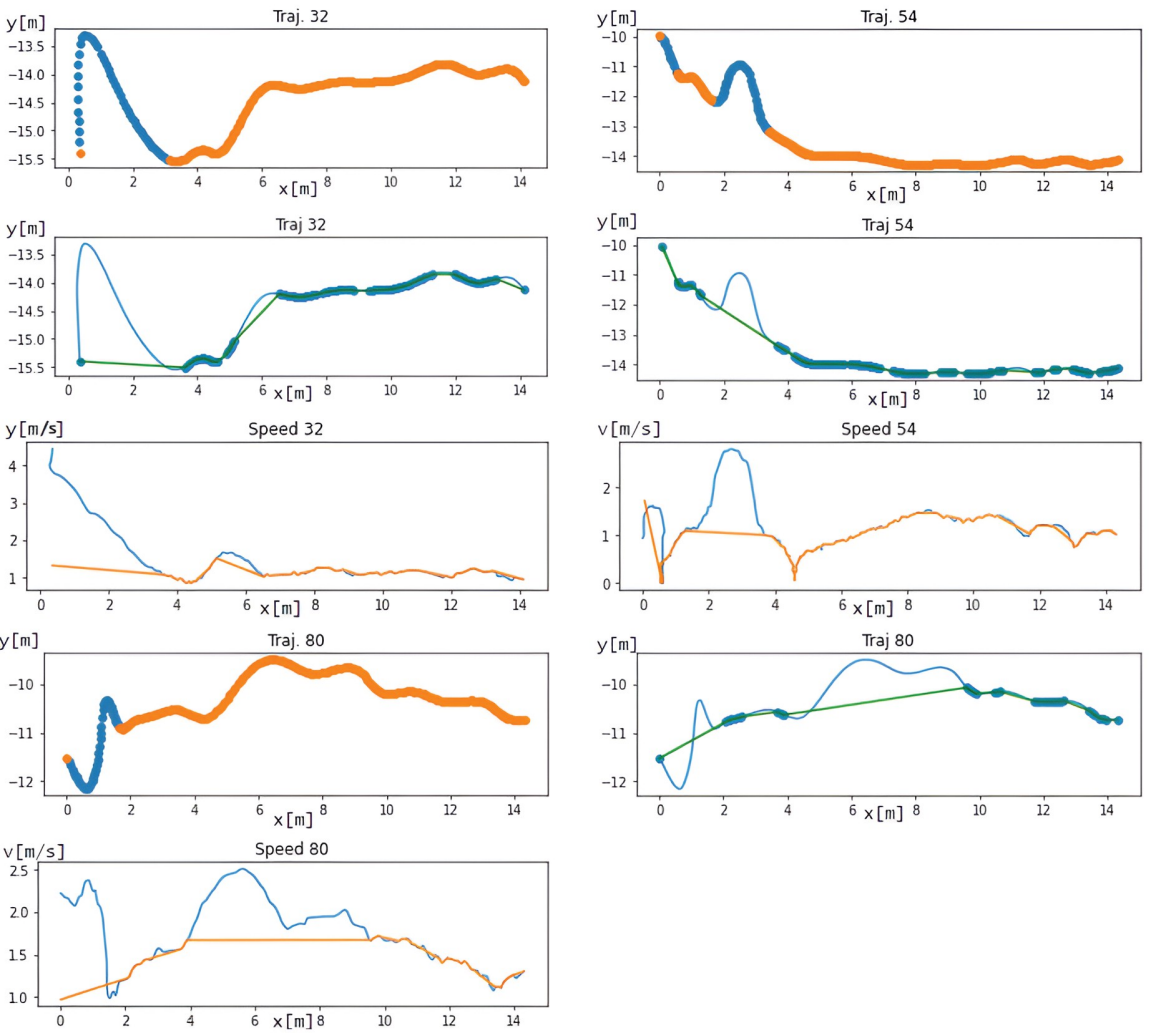
View corrected trajectories from the test dataset (left column) and their comparison with manually defined erroneous points (right column). The green curve indicates the corrected trajectory.

The following Fig 16 shows the trajectories from the test set. New points are created by linear interpolation because the analysis shows that noise segments are areas where the straight segment is replaced by a deflection. These new segments could be replaced in a more complex way, such as predicting them using an LSTM neural network.

### Agent-based model calibration

The model of the vaccination center waiting room was designed in Pathfinder software (version 2022.2.0803) [5] in two settings:

- Model A—basic, where input flow and walking speed are calibrated and other settings of agents’ behavior correspond to default Pathfinder settings; this setup corresponds to a situation when there is no data about occupants’ behavior in the waiting room.
- Model B—advanced, where walking speed, location and distribution of waiting points, waiting time, and exit selection are calibrated; this setting corresponds to use of calibration parameters extracted from trajectories detected by pedestrian traffic detector.

Table 4 describes parameters used for model calibration in Pathfinder. Waiting was defined as when instantaneous walking speed is less than 0.4 m.s^-1^ or 0.4 m.s^-1^ and distance from position one second ago is less than 0.4 m. Analysis shows that 40% of occupants (90 occupants) walked straight through waiting room and 60% (136 occupants) stopped at least once. Based on a 2D kernel Gaussian probability density function, four waiting points were estimated (see Fig 18). 78.8% of occupants (107 occupants) made one stop and 21.2% of occupants (29 occupants) made two stops while passing through waiting room. For all waiting points, distribution of their use by persons and waiting time is also determined. Results of walking speed analysis showed normal distribution with parameters mu: 1.17 m.s^-1^ and sigma: 0.21 m.s^-1^.

**Table 4:** Model parameters used for calibration.

Parameter	Values
**Distribution of waiting (at least once) and walking**	waiting 60% / walking 40%
**Waiting point 1 location (WP1)**	x: 1.34, y: -15.5
**Waiting point 2 location (WP2)**	x: 12.09, y: -13.73
**Waiting point 3 location (WP3)**	x: 0.88, y: -11.18
**Waiting point 4 location (WP4)**	x: 4.49, y: -14.26
**Number of stops distribution**	1 stop: 78.8%, 2 stops: 21.2%
**Distribution of waiting points for 1/1 waiting**	WP1: 3.85%, WP2: 39.42%, WP3: 34.62%, WP4: 22.11%
**Distribution of waiting points for 1/2 waiting**	WP1: 7.14%, WP2: 25%, WP3: 3.57%, WP4: 64.29%
**Distribution of waiting points for 2/2 waiting**	WP1: 39.29%, WP2: 0%, WP3: 42.86%, WP4: 17.85%
**Walking speed [m/s]–normal distribution**	min: 0.82, μ: 1.17, σ: 0.21, max: 1.52
**Exit choice distribution**	exit 1: 61%, exit 2: 39%

**Fig 17:**
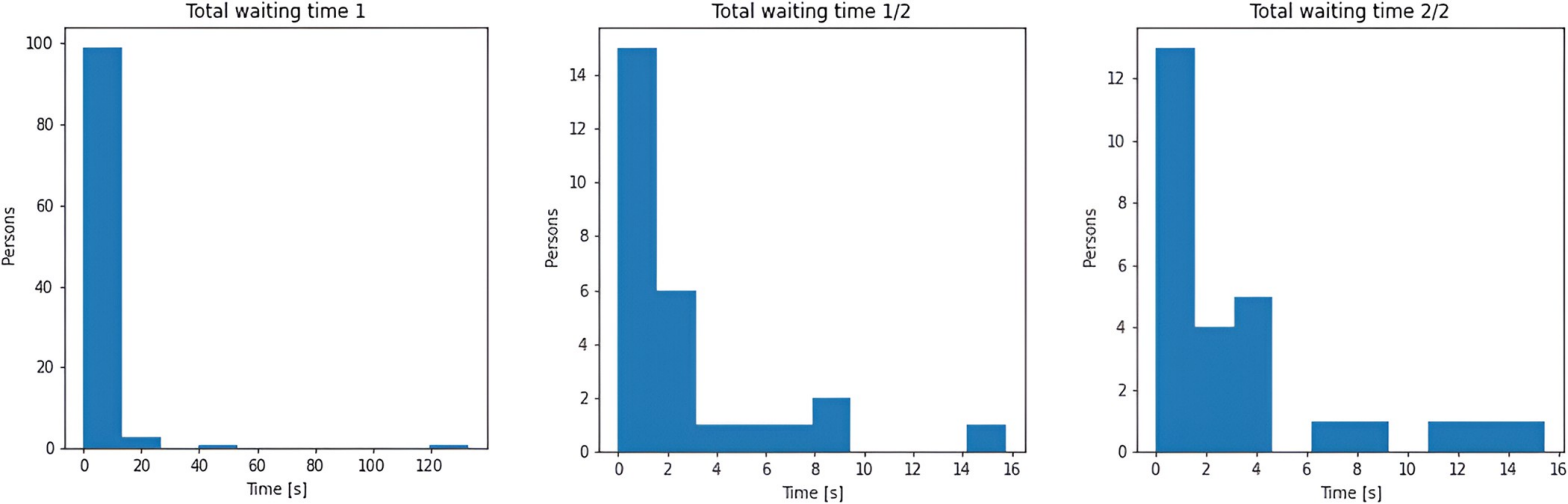
Waiting times for 1/1 waiting (left), 1/2 waiting (middle), 2/2 waiting (right).

Waiting times at particular waiting points is described by histograms at Fig 17. Most of waiting times lies between 0–10 seconds.

Based on the stochasticity of the Pathfinder model, the number of simulations run for both settings was set to 20 runs. The number of runs was determined based on Ronchi’s method [42].

### Agent-based model validation

**Fig 18:**
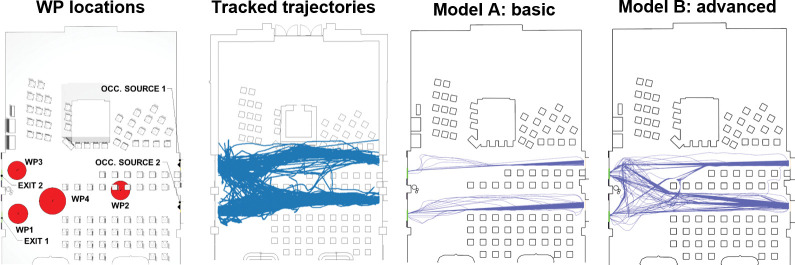
Waiting points location and qualitative validation of trajectory shapes of cleaned trajectories from pedestrian traffic detector, model A–basic, and model B–advanced.

To demonstrate the use of the proposed calibration procedure, only one qualitative validation was used to visually compare the trajectory bundle. Other quantitative metrics implemented are not mentioned in this paper. A visual comparison of the shape of the trajectories was performed based on the Fig 18. Data from one simulation per each model was used for this purpose. The figure shows that the introduction of behavioral parameters into the model led to an overall refinement of the shape of the trajectories according to the trajectories from the pedestrian traffic detector.

## Conclusion

Currently, it is possible to use a large amount of CCTV data to calibrate pedestrian models. In this paper, we consider extracting model parameters from trajectories obtained from an automatic pedestrian traffic detector and subsequently calibrating a vaccination center waiting room model. The parameter extraction involved DBScan cluster analysis for automatic filtering of client and staff trajectories. The Random Forest classification algorithm was used to detect erroneous points in the trajectories caused by occlusion. Based on the cleaned data, the parameters of the normal distribution of walking speed, waiting points distribution, waiting points location, waiting times distribution, and exit choice distribution were calculated. The model was designed in two settings—basic, i.e., with calibrated input flow and walking speed only, and advanced—with calibration of all extracted parameters. A visual validation test was performed to evaluate the shape of trajectories in simulation. The results showed a significantly better fit of the advanced model compared to the baseline model against the cleaned CCTV data.

The proposed calibration procedure allows testing the effect of procedural changes on the throughput of the vaccination center or other facilities—e.g., changes in the form of registration of clients of the vaccination center, changes in the number of staff in a given section of the facility, number of administrative and medical tasks, or vaccination settings (optimal number of patients seen in parallel)—which can have a significant impact on system throughput. A limitation is that in parts of the facility where behavioral parameters have been calibrated, geometric changes to the facility cannot be tested as this could result in significantly different behavior of occupants—different waiting areas and waiting times. Therefore, testing geometric modifications can be performed in parts of the facility that are adjacent or preceding the part where behavioral parameters are calibrated. As a result, the effect of procedural changes described above on facility throughput can be tested as a function of different geometric arrangements of specific parts of the facility. Changes in input intensity of flow of occupants in front of calibrated elements can also be problematic and lead to significantly different behavior. To verify capacity and identify bottleneck of whole system, it is always necessary to extract model parameters from camera data taken at peak occupancy of facility.

Another shortcoming is the instability of the algorithm when there is a high density of occupants, where a person is often lost and the trajectory becomes confused. In such situations, it can be very difficult for an algorithm to reliably identify and track individuals, and their trajectories can become confused or even lost altogether. The proposal of a reliable algorithm for detecting and tracing occupants in a crowd from tracked trajectories is a frequent topic of research.

As the field of machine learning continues to advance, there is an increasing demand for more efficient and effective methods of predicting model output values in real time during the operation of the object under study. This is particularly important in scenarios where quick and accurate decision-making is crucial, such as in industrial control systems, autonomous vehicles, and medical diagnosis. Further research in this area will focus on developing novel algorithms and techniques that can automatically predict model output values in real time, based on data generated by the object under study. This will involve the use of advanced statistical and machine learning methods, such as deep learning, reinforcement learning, and Bayesian inference.

One potential solution to this problem is the creation of adaptive agents, which can adjust their behavior based on data extracted from the model. These agents can be trained using data generated by the object under study and can use this data to dynamically adjust their properties and decision-making processes. This approach has the potential to significantly improve the accuracy and efficiency of real-time model predictions, as well as enable more sophisticated control systems and intelligent agents. Overall, the development of automated real-time prediction methods and adaptive agents based on data extracted from models has the potential to revolutionize a wide range of fields, from manufacturing and logistics to healthcare and finance. As such, it is an exciting area of research that is likely to see significant progress in the coming years.
